# A critical role for Th17 cell-derived TGF-β1 in regulating the stability and pathogenicity of autoimmune Th17 cells

**DOI:** 10.1038/s12276-021-00632-9

**Published:** 2021-05-28

**Authors:** Garam Choi, Young-Jun Park, Minkyoung Cho, Heesu Moon, Daehong Kim, Chang-Yuil Kang, Yeonseok Chung, Byung-Seok Kim

**Affiliations:** 1 Laboratory of Immune Regulation, Research Institute of Pharmaceutical Sciences, College of Pharmacy, Seoul National University, Seoul, 08826 Republic of Korea; 2 BK21 Plus Program, College of Pharmacy, Seoul National University, Seoul, 08826 Republic of Korea; 3 Department of Pharmacy, College of Pharmacy, Jeju National University, Jeju, Jeju Special Self-Governing Province, 63243 Republic of Korea; 4 Interdisciplinary Graduate Program in Advanced Convergence Technology and Science, Jeju National University, Jeju, Jeju Special Self-Governing Province, 63243 Republic of Korea; 5 Laboratory of Immunology, Research Institute of Pharmaceutical Sciences, College of Pharmacy, Seoul National University, Seoul, 08826 Republic of Korea; 6 Division of Life Sciences, College of Life Science and Bioengineering, Incheon National University, Incheon, 22012 Republic of Korea

**Keywords:** Autoimmunity, Neuroimmunology

## Abstract

Pathogenic conversion of Th17 cells into multifunctional helper T cells or Th1 cells contributes to the pathogenesis of autoimmune diseases; however, the mechanism regulating the plasticity of Th17 cells remains unclear. Here, we found that Th17 cells expressed latent TGF-β1 in a manner dependent on autocrine TGF-β1. By employing IL-17-producing cell-specific *Tgfb1* conditional knockout and fate-mapping systems, we demonstrated that TGF-β1-deficient Th17 cells are relatively susceptible to becoming IFN-γ producers through IL-12Rβ2 and IL-27Rα upregulation. TGF-β1-deficient Th17 cells exacerbated tissue inflammation compared to TGF-β1-sufficient Th17 cells in adoptive transfer models of experimental autoimmune encephalomyelitis and colitis. Thus, TGF-β1 production by Th17 cells provides an essential autocrine signal for maintaining the stability and regulating the pathogenicity of Th17 cells in vivo.

## Autoimmune disease: Transforming growth factor keeps subset of T cells in check

A protein secreted by pro-inflammatory immune cells acts on the same secreting cells to prevent their conversion into pathogenic drivers of autoimmune disease. A team in South Korea led by Byung-Seok Kim from Incheon National University and Yeonseok Chung from Seoul National University showed that T helper 17 (Th17) cells, a subset of T helper cells defined by their production of a signaling molecule known as interleukin 17, express a protein called transforming growth factor-β1 (TGF-β1) that maintains the stability of Th17 in a self-regulating manner. Without the ability to produce TGF-β1, the cells tend to convert into a form that fuel disease-associated inflammation in mouse models of multiple sclerosis and colitis. Therapeutic blockade of this conversion process could help treat diseases linked to Th17 cell–mediated autoimmunity.

## Introduction

Although IL-17-producing T helper cells (Th17 cells) have an essential role in mediating host protection against extracellular pathogens, they also mediate the development of autoimmune diseases, including multiple sclerosis, inflammatory bowel diseases, rheumatoid arthritis, and psoriasis, in humans^1–3^. Early Th17 cell differentiation is initiated by stimulation with transforming growth factor-β1 (TGF-β1) in combination with IL-6, which induces the expression of STAT3 and the lineage-determining transcription factor RORγt. In addition, IL-1 and IL-23 signals are required for differentiating Th17 cells to complete the lineage commitment program and drive autoimmune inflammation^4,5^.

Unlike Th1 and Th2 cells, Th17 cells are known to be plastic: they can transdifferentiate into other Th subsets, particularly under lymphopenic or inflammatory conditions^6,7^. Of all the known processes involving Th17 cells transdifferentiating into various effector cell types, including follicular helper T cells and regulatory T (Treg) cells, under inflammatory conditions^8,9^, conversion of Th17 cells into IL-17^+^IFN-γ^+^ cells (Th1-like Th17 cells) or IL-17^-^IFN-γ^+^ cells (Th1-like exTh17 cells) has been recognized as one of the major and critical pathways of Th17 cell plasticity during the development of autoimmune diseases in mice and humans^2,6^. Fate mapping of IL-17-producing cells provided strong evidence that most myelin-reactive Th1 cells originate from Th17 cells in experimental autoimmune encephalomyelitis (EAE), although the importance of Th17 cell-derived Th1 cell populations in the pathogenesis of autoimmune diseases remains elusive^6,10–12^. Previous studies identified several molecular pathways, such as PTEN, mTOR, and Notch1/RBPJ signaling pathways, that regulate the pathogenic conversion of Th17 cells into Th1 cells in autoimmunity^13–15^. In addition, a recent study demonstrated that cell-surface expression of Fas on Th17 cells inhibits Th17 cell conversion into Th1 cells by controlling STAT1 activation^16^. However, the detailed mechanism regulating the stability of autoimmune Th17 cells remains unclear.

TGF-β1 is a pleiotropic immunomodulatory cytokine that plays a pivotal role in immune homeostasis^17^. While more than 90% of circulating TGF-β1 is produced by platelets in the steady state, multiple cell types, including Treg cells and γδ T cells, also produce TGF-β1^18^. A few studies have reported that TGF-β1 is essential for the differentiation of Th17 cells^19,20^, whereas others have suggested TGF-β1-independent induction of pathogenic Th17 cells^4,21^. While initial studies showed that TGF-β1 from Treg cells critically contributes to Th17 cell differentiation^22^, autocrine TGF-β1 appears to be essential for Th17 cell differentiation in vivo^23^. In addition to IL-6 and TGF-β1, IL-1β and IL-23 are required for the terminal differentiation of initially committed Th17 cells by repressing IL-10, inducing the expression of Blimp-1 in Th17 cells^4,24,25^. However, repetitive stimulation with IL-23 is known to gradually deprive Th17 cells of IL-17 while upregulating IFN-γ expression^26^. Thus, unlike the initial differentiation program, the mechanism that maintains the stability of differentiated Th17 cells in vivo needs to be addressed in further studies. In this study, we aimed to investigate the molecular mechanism governing the stability and plasticity of Th17 cells in vivo. We found that Th17 cell-derived TGF-β1 plays a crucial role not only in the expression of latent TGF-β1 on Th17 cells but also in the maintenance of Th17 cell stability by repressing the expression of IL-12Rβ2 and IL-27Rα. Consequently, TGF-β1-deficient Th17 cells appeared to be more susceptible to conversion into pathogenic Th1-like Th17 cells and Th1-like exTh17 cells, resulting in exacerbated inflammation in the central nervous system (CNS) or the intestine in vivo.

## Results

### Regulation of latent TGF-β1 expression in Th17 cells

**Fig. 1:**
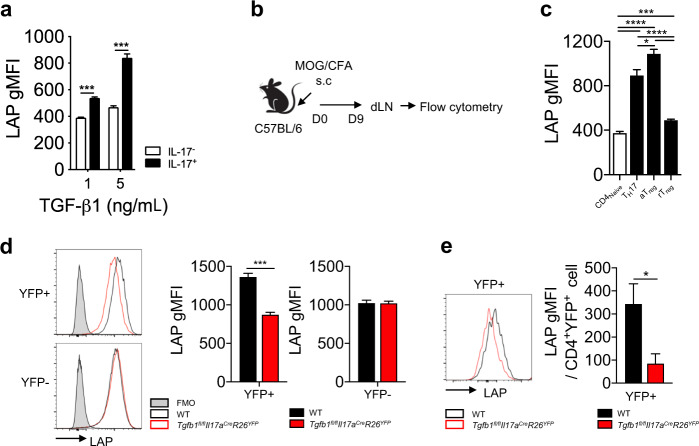
Th17 cell-derived TGF-β1 is crucial for LAP expression in Th17 cells. **a** Naive CD4^+^ T cells were differentiated into Th17 cells with IL-6 and TGF-β1 for 3 days. Geometric mean fluorescence intensities (gMFIs) of LAP in gated IL-17^+^ or IL-17^−^ cells were examined. **b** Experimental procedure. The indicated mice were subcutaneously (s.c.) immunized with myelin oligodendrocyte glycoprotein peptide (MOG_35-55_) in complete Freund’s adjuvant (CFA). Nine days after immunization, lymphocytes from the draining lymph nodes (dLNs) were analyzed by flow cytometry. **c** gMFIs of LAP in CD4^+^CD3ε^+^Foxp3^-^CD44^lo^ T (CD4_Naive_), IL-17^+^CD4^+^CD44^hi^ T (T_H_17), CD4^+^Foxp3^+^CD44^hi^ T (activated Treg, aTreg) and CD4^+^Foxp3^+^CD44^lo^ T (resting Treg, rTreg) cells from the dLNs were measured (*n* = 5). **d** Naive CD4^+^ T cells from *Tgfb1*^*fl/fl*^*Il17a*^*Cre*^*R26*^*YFP*^ or wild-type (WT, *Il17a*^*Cre*^*R26*^*YFP*^ or *Tgfb1*^*fl/+*^*Il17a*^*Cre*^*R26*^*YFP*^) mice were differentiated into Th17 cells and analyzed for the expression of LAP. Representative histogram and gMFIs of LAP in fluorescence minus one (FMO) control and gated YFP^+^ or YFP^−^ cells are shown. **e** *Tgfb1*^*fl/fl*^*Il17a*^*Cre*^*R26*^*YFP*^ and WT mice were immunized with MOG_35-55_ peptide in CFA. After 8–9 days, lymphoid cells were obtained from the dLNs and restimulated with the MOG_35-55_ peptide in the presence of IL-23 and an anti-IFN-γ antibody for 5 days to enrich MOG_35-55_-specific Th17 cells. Representative histogram and gMFIs of LAP in gated CD4^+^YFP^+^ cells are shown. Data are representative of three independent experiments, and values are expressed as the mean + SEM (**a**, **c**, **d**, and **e**); *p < 0.05, ***p < 0.001 and ****p < 0.0001. A two-tailed Student’s *t*-test was performed.

While the critical contributions of autocrine and paracrine TGF-β1 produced by activated CD4^+^ T cells or Treg cells to the generation of Th17 cells is well established^23^, it remains unclear whether TGF-β1 plays any role in the maintenance of already committed Th17 cells. As a first step to exploring the role of TGF-β1 in the maintenance of Th17 cells, we examined the expression of latent TGF-β1 in Th17 cells differentiated with IL-6 and TGF-β1. The expression of latency-associated peptide (LAP), a dimeric pro-peptide associated with dimeric mature TGF-β1, was higher in the IL-17^+^ population than in the IL-17^*−*^ population (Fig. 1a). To confirm the expression of LAP in Th17 cells generated in vivo, we compared the expression level of LAP among naïve CD4^+^ T, Th17, and Treg cells in the draining lymph nodes (dLNs) of mice immunized with a myelin oligodendrocyte glycoprotein peptide (MOG_35-55_) in complete Freund’s adjuvant (CFA) (Fig. 1b). Notably, the expression level of LAP in Th17 cells appeared to be significantly higher than that in CD44^lo^ naïve CD4^+^ T cells and CD44^lo^ resting Treg cells but slightly lower than that in CD44^hi^ activated Treg cells (Fig. 1c). By analyzing the gene expression profiles of human CD4^+^ T cell subsets^27^, we found that human Th17 cells highly expressed *TGFB1* compared to naïve CD4^+^ T cells (Fig. S1).

These observations, together with a recent study suggesting the production of TGF-β1 by Th17 cells^23^, raised the possibility that Th17 cell-derived TGF-β1 plays a role in LAP expression in Th17 cells. To address this possibility, we generated mice conditionally deficient in TGF-β1 in IL-17-producing cells by crossing *Il17a-Cre* mice with *Tgfb1*-floxed mice (*Tgfb1*^*fl/fl*^*Il17a*^*Cre*^). To enable fate mapping (fm) of IL-17-producing cells, *Tgfb1*^*fl/fl*^*Il17a*^*Cre*^ mice were further crossed with *R26*^*YFP*^ mice, in which Cre recombinase-expressing cells are permanently labeled with yellow fluorescent protein (YFP). We next compared the expression of LAP between the IL-17^+^ and IL-17^*−*^ populations of CD4^+^ T cells stimulated under Th17-skewing conditions in vitro. While the expression of LAP was comparable in IL-17fm-YFP^-^ cells between *Tgfb1*^*fl/fl*^*Il17a*^*Cre*^*R26*^*YFP*^ mice and wild-type (WT, *Il17a*^*Cre*^*R26*^*YFP*^ or *Tgfb1*^*fl/+*^*Il17a*^*Cre*^*R26*^*YFP*^*)* mice (Fig. 1d), it was decreased in IL-17fm-YFP^+^ cells from *Tgfb1*^*fl/fl*^*Il17a*^*Cre*^*R26*^*YFP*^ mice compared to those from WT. The reduction in LAP expression in T cells from *Tgfb1*^*fl/fl*^*Il17a*^*Cre*^*R26*^*YFP*^ mice was selective for Th17 cells since we did not observe any defect in LAP expression in Th1 or induced Treg (iTreg) cells (Fig. S2a and b). Consistently, we also observed a significantly decreased level of LAP expression in IL-17fm-YFP^+^ cells from *Tgfb1*^*fl/fl*^*Il17a*^*Cre*^*R26*^*YFP*^ mice compared to IL-17fm-YFP^+^ cells from control mice generated in vivo after immunization with MOG_35-55_ peptide in CFA (Fig. 1e). These results indicate that Th17 cells produce TGF-β1 and that Th17-derived TGF-β1 is required for the expression of latent TGF-β1 in Th17 cells.

### Th17 cell-derived TGF-β1 maintains the stability of Th17 cells

**Fig. 2:**
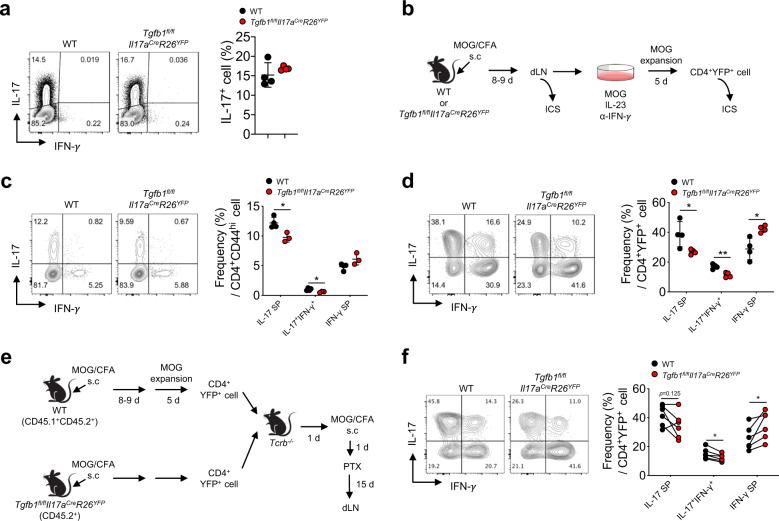
TGF-β1 produced by Th17 cells is required for the stability of Th17 cells. **a** Naive CD4^+^ T cells from *Tgfb1*^*fl/fl*^*Il17a*^*Cre*^*R26*^*YFP*^ or WT mice were differentiated into Th17 cells for 3 days and analyzed for the expression of cytokines by flow cytometry. Representative FACS plots and quantification of IFN-γ- and/or IL-17-expressing cells. **b** Experimental scheme. *Tgfb1*^*fl/fl*^*Il17a*^*Cre*^*R26*^*YFP*^ and WT mice were immunized with MOG_35-55_ peptide in CFA. Eight to nine days after immunization, lymphocytes from the dLNs were stimulated with MOG_35-55_ peptide in the presence of IL-23 and an anti-IFN-γ antibody. After 5 days, the expression of cytokines was determined by flow cytometry. **c** Representative contour plots and quantification of IFN-γ- and/or IL-17-expressing cells (IL-17^+^IFN-γ^−^: IL-17 SP, IL-17^−^IFN-γ^+^: IFN-γ SP) in CD4^+^CD44^hi^ cells from the indicated mice. **d** Representative FACS plots and quantification of IFN-γ- and/or IL-17-expressing cells in CD4^+^YFP^+^ cells. **e** Schematic representation of the T cell adoptive cotransfer experimental autoimmune encephalomyelitis (EAE) model. *Tgfb1*^*fl/fl*^*Il17a*^*Cre*^*R26*^*YFP*^ (CD45.2^+^) and WT (CD45.1^+^CD45.2^+^) mice were immunized with MOG_35-55_ peptide in CFA. After 8–9 days, lymphocytes from the dLNs were stimulated with MOG_35-55_ peptide in the presence of IL-23 and an anti-IFN-γ antibody. After 5 days of stimulation, CD4^+^YFP^+^ T cells were sorted on a FACSAria^TM^ III, mixed at a 1:1 ratio and adoptively transferred into *Tcrb*^*−/−*^ mice. Recipient mice were immunized with MOG_35-55_/CFA and intraperitoneally injected with pertussis toxin (PTX) (*n* = 6). **f** Representative contour plots and quantification of IFN-γ- and/or IL-17-expressing cells in CD4^+^YFP^+^ cells from the dLNs after restimulation with PMA and ionomycin (*n* = 6). Data are representative of three independent experiments. Quantification plots show the mean ± SD (**a**, **c**, and **d**); *p < 0.05 and **p < 0.01. A two-tailed Student’s *t*-test (**c** and **d**) and the Wilcoxon signed-rank test (**f**) were performed.

We next sought to determine the roles of Th17 cell-derived TGF-β1 in the differentiation and maintenance of Th17 cells. When naïve CD4^+^ T cells from *Tgfb1*^*fl/fl*^*Il17a*^*Cre*^*R26*^*YFP*^ mice or control mice were differentiated under Th17 cell-polarizing conditions in vitro, we did not observe any significant difference in the frequency of Th17 cells (Fig. 2a). To determine the roles of Th17 cell-derived TGF-β1 in the generation and expansion of antigen-specific Th17 cells, *Tgfb1*^*fl/fl*^*Il17a*^*Cre*^*R26*^*YFP*^ mice and control mice were immunized with MOG_35-55_ peptide emulsified in CFA (Fig. 2b). We found that the frequency of IL-17fm-YFP^+^ cells in the dLNs was comparable between the two groups, indicating that Th17 cell-derived TGF-β1 played a minor role in initial Th17 cell differentiation in vivo (Fig. S3a). In contrast, the frequencies of IL-17^+^IFN-γ^−^ cells and IL-17^+^IFN-γ^+^ cells within the CD4^+^CD44^hi^ T cell population were slightly but significantly lower in *Tgfb1*^*fl/fl*^*Il17a*^*Cre*^*R26*^*YFP*^ mice than in control mice (Fig. 2c). To determine the role of Th17 cell-derived TGF-β1 in the maintenance of antigen-specific Th17 cells, we stimulated total lymphocytes from the dLNs with the MOG_35-55_ peptide in the presence of an anti-IFN-γ antibody and IL-23. On day 5, the frequencies of IL-17^+^IFN-γ^−^ cells and IL-17^+^IFN-γ^+^ cells were significantly lower in TGF-β1-deficient YFP^+^ cells than in TGF-β1-sufficient YFP^+^ cells (Fig. 2d). However, TGF-β1-deficient YFP^+^ cells contained more IL-17^-^IFN-γ^+^ Th1-like exTh17 cells than did TGF-β1-sufficient YFP^+^ cells.

To determine whether the increase in the IL-17^-^IFN-γ^+^ cell subpopulation in TGF-β1-deficient Th17 cells was due to the deficiency in autocrine TGF-β1, we adoptively transferred a 1:1 mixture of MOG_35-55_-specific TGF-β1-sufficient IL-17fm-YFP^+^ cells (CD45.1^+^CD45.2^+^) and TGF-β1-deficient IL-17fm-YFP^+^ cells (CD45.2^+^) into TCRβ-deficient recipient mice before immunizing the recipients with the MOG_35-55_ peptide in CFA and subsequently injecting pertussis toxin (PTX) (Fig. 2e). The recipient mice developed typical clinical symptoms of EAE, such as loss of tail tonicity and hindlimb paralysis. Consistent with the results in Fig. 2d, we observed that the CD45.1^−^ TGF-β1-deficient IL-17fm-YFP^+^ cells contained an increased subpopulation of IL-17^-^IFN-γ^+^ cells but decreased subpopulations of IL-17^+^IFN-γ^+^ and IL-17^+^IFN-γ^−^ cells compared to the CD45.1^+^ control IL-17fm-YFP^+^ cells in the dLNs and CNS (Fig. 2f and S3b). Taken together, these ex vivo and in vivo results demonstrate that Th17 cell-derived TGF-β1 is dispensable for the generation of Th17 cells but is required for the stability and maintenance of Th17 cells.

### Autocrine TGF-β1 regulates IL-12Rβ2 and IL-27Rα expression in Th17 cells

**Fig. 3:**
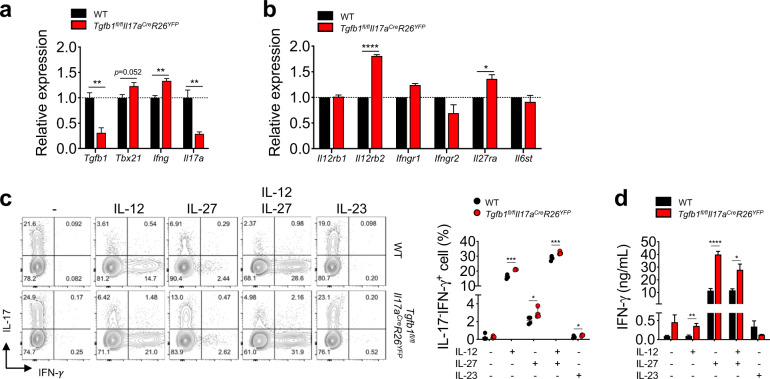
Th17 cell-specific deletion of TGF-β1 induces conversion of Th17 cells into IFN-γ-producing cells by IL-12 and IL-27. **a**, **b** *Tgfb1*^*fl/fl*^*Il17a*^*Cre*^*R26*^*YFP*^ and WT mice were immunized with MOG_35-55_ peptide in CFA. Eight to nine days after immunization, lymphocytes isolated from the dLNs were stimulated with MOG_35-55_ peptide in the presence of IL-23 and an anti-IFN-γ antibody to enrich myelin-reactive Th17 cells. After 5 days, CD4^+^YFP^+^ cells were sorted, and relative gene expression was analyzed by qRT-PCR. **a** Relative expression of *Tgfb1, Tbx21, Ifng*, and *Il17a*. **b** The relative gene expression of cytokine receptors in myelin-reactive Th17 cells was analyzed by qRT-PCR. **c**, **d** Th17 cells differentiated from naïve CD4^+^ T cells were stimulated with anti-CD3ε and anti-CD28 antibodies in the presence or absence of cytokines (IL-12, IL-27, or IL-23) for 3 days and analyzed by flow cytometry and ELISA. **c** Representative contour plots and quantification of IFN-γ- and/or IL-17-expressing cells in YFP^+^CD4^+^ cells. **d** Quantification of IFN-γ in the supernatant of stimulated Th17 cells after 3 days of culture. Data are representative of three independent experiments. Quantification plots show the mean + SEM (**a**, **b**, and **d**) and ± SD (**c**); *p < 0.05, **p < 0.01, ***p < 0.001, and ****p < 0.0001. A two-tailed Student’s *t*-test was performed.

To investigate the molecular mechanism by which autocrine TGF-β1 contributes to the stability of Th17 cells, we comparatively analyzed the expression levels of genes related to the Th1 cell differentiation program in myelin-reactive IL-17fm-YFP^+^ Th17 cells from *Tgfb1*^*fl/fl*^*Il17a*^*Cre*^*R26*^*YFP*^ and WT mice. As expected, the level of *Tgfb1* was significantly diminished in IL-17fm-YFP^+^ cells from *Tgfb1*^*fl/fl*^*Il17a*^*Cre*^*R26*^*YFP*^ mice compared to those from control mice (Fig. 3a). In accordance with the observed elevation in IFN-γ expression, TGF-β1-deficient IL-17fm-YFP^+^ cells exhibited increased expression of *Tbx21* and *Ifng* (Fig. 3a). In contrast, the expression level of *Il17a* was decreased in TGF-β1-deficient IL-17fm-YFP^+^ cells compared to TGF-β1-sufficient IL-17fm-YFP^+^ cells. Among the genes for Th1-associated cytokine receptors, we observed that the expression of *Il12rb2* and *Il27ra* in TGF-β1-deficient IL-17fm-YFP^+^ cells was significantly higher than that in TGF-β1-sufficient IL-17fm-YFP^+^ cells, while the expression of *Il12rb1*, *Ifngr1*, *Ifngr2*, and *Il6st* remained comparable between the two groups (Fig. 3b). These data suggest that TGF-β1-deficient IL-17fm-YFP^+^ cells are more likely to be sensitive to IL-12 and IL-27 signals due to the increased expression of the corresponding receptors. To test this hypothesis, we employed a Th17 cell conversion assay in which fully differentiated Th17 cells were restimulated with TCR signaling in the presence of an additional cytokine(s). To this end, TGF-β1-sufficient or TGF-β1-deficient IL-17fm-YFP^+^ cells purified from in vitro Th17 cultures were restimulated with anti-CD3ε and anti-CD28 antibodies in the presence of IL-12, IL-27, or IL-23 for 3 days, and then the expression of IL-17 and IFN-γ was analyzed. While stimulation with the anti-CD3ε and anti-CD28 antibodies did not induce IL-17^-^IFN-γ^+^ cells, exogenous IL-12 or IL-27 alone significantly induced IL-17^-^IFN-γ^+^ cells in the IL-17fm-YFP^+^ cell cultures, and this population was synergistically increased by combination treatment with IL-12 and IL-27 (Fig. 3c). In contrast, the effect of IL-23 on the induction of IL-17^-^IFN-γ^+^ cells in the IL-17fm-YFP^+^ cell cultures was marginal. Of note, the frequency of IL-17^-^IFN-γ^+^ cells was uniformly increased in TGF-β1-deficient IL-17fm-YFP^+^ cells compared to TGF-β1-sufficient IL-17fm-YFP^+^ cells in the presence of IL-12 or IL-27, indicating that TGF-β1-deficient IL-17fm-YFP^+^ cells are more sensitive to IL-12 and IL-27, presumably due to the increased expression of IL-12Rβ2 and IL-27Rα, respectively. While the frequency of IL-17^-^IFN-γ^+^ cells was significantly increased in TGF-β1-deficient IL-17fm-YFP^+^ cells even in the presence of both IL-12 and IL-27, the difference between TGF-β1-sufficient IL-17fm-YFP^+^ cells and TGF-β1-deficient IL-17fm-YFP^+^ cells was relatively marginal, likely because the combination of these two cytokines might have overridden the effect of autocrine TGF-β1 on Th17 cell stability. Consistent with the increased frequencies of IL-17^-^IFN-γ^+^ cells, we observed significantly increased amounts of IFN-γ in the culture supernatant of TGF-β1-deficient IL-17fm-YFP^+^CD4^+^ T cells in response to IL-12 or IL-27 stimulation (Fig. 3d). Interestingly, however, more IFN-γ was detected in the culture supernatant of IL-27-stimulated Th17 cells than in that of IL-12-stimulated Th17 cells regardless of the TGF-β1-producing capacity of the Th17 cells (Fig. 3d). Overall, these results demonstrate an essential role for autocrine TGF-β1 in the maintenance of Th17 cell stability via repression of both IL-12- and IL-27-dependent IFN-γ expression in Th17 cells.

### Autocrine TGF-β1 inhibits the pathogenic conversion of Th17 cells into Th1-like exTh17 cells in EAE and experimental colitis models

**Fig. 4:**
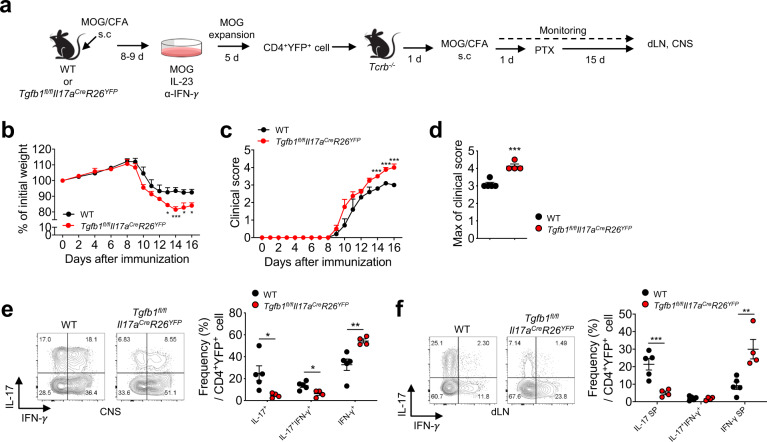
Autocrine TGF-β1 regulates the pathogenicity of myelin-reactive Th17 cells. **a** Schematic representation of the T cell transfer EAE model. *Tgfb1*^*fl/fl*^*Il17a*^*Cre*^*R26*^*YFP*^ and WT mice were immunized with MOG_35-55_ peptide in CFA. Eight to nine days after immunization, lymphocytes isolated from the dLNs were stimulated with MOG_35-55_ peptide in the presence of IL-23 and an anti-IFN-γ antibody. After 5 days of stimulation, FACS-sorted CD4^+^YFP^+^ T cells were adoptively transferred into *Tcrb*^*−/−*^ mice, and the recipient mice were intraperitoneally injected with PTX following MOG_35-55_/CFA immunization. **b** The percentage weight change relative to body weight on day 0 is shown (*n* = 4–5). **c**, **d** Clinical score (**c**) and maximum clinical score (**d**) are shown (*n* = 4–5). **e** Representative FACS plots and quantification of IFN-γ- and/or IL-17-expressing cells in the CD4^+^YFP^+^ cell population in the central nervous system (CNS). **f** Representative FACS plots and quantification of IFN-γ- and/or IL-17-expressing cells in the CD4^+^YFP^+^ cell population in the dLNs. Data are representative of two independent experiments. Quantification plots show the mean + SEM (**b** and **c**) and ± SD (**d**–**f**); *p < 0.05, **p < 0.01, and ***p < 0.001. A two-tailed Student’s *t*-test was performed.

We next sought to examine the role of Th17-derived TGF-β1 in the pathogenicity of Th17 cells in the autoimmune setting by adoptively transferring myelin-reactive TGF-β1-sufficient or TGF-β1-deficient Th17 cells into *Tcrb*^*−/−*^ mice (Fig. 4a). To our surprise, the recipients of TGF-β1-deficient Th17 cells exhibited worse symptoms, as indicated by accelerated weight loss, increased clinical severity, and a higher maximum clinical score, than those of TGF-β1-sufficient Th17 cells (Fig. 4b–d). To investigate whether the increased disease severity was associated with decreased stability of TGF-β1-deficient Th17 cells, we assessed the production of IFN-γ and IL-17 by CD4^+^YFP^+^ cells in the CNS and dLNs of the recipients. The transferred TGF-β1-deficient Th17 cells recovered from the inflamed CNS and dLNs displayed a significantly reduced frequencies of IL-17^+^IFN-γ^−^ and IL-17^+^IFN-γ^+^ cells and an increased frequency of IL-17^−^IFN-γ^+^ cells compared to the transferred TGF-β1-sufficient Th17 cells (Fig. 4e and f).

**Fig. 5:**
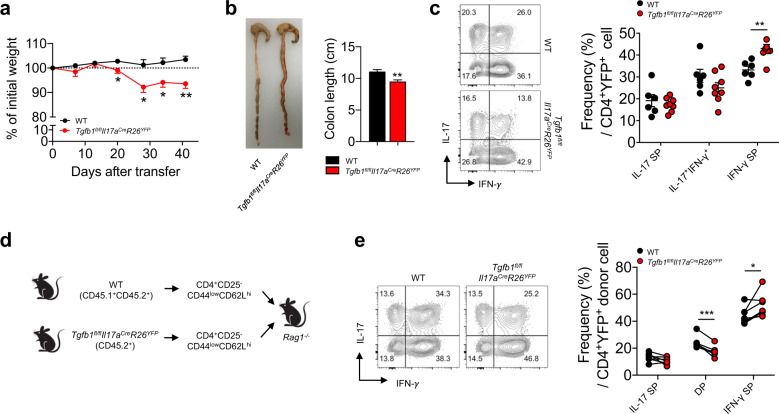
Autocrine TGF-β1 inhibits CD4^+^ T cell-mediated intestinal inflammation. **a**–**c** Naïve CD4^+^ T cells were sorted and adoptively transferred into *Rag1*^*−/−*^ mice. **a** Recipient mice were monitored weekly for weight loss, and the body weight change (% of initial weight) was calculated (*n* = 6–8). **b** Representative pictures of colons and the mean colon length are shown. **c** IFN-γ and IL-17 expression in CD4^+^YFP^+^ cells in the mesenteric LNs of recipient mice and the frequencies of IFN-γ- and/or IL-17-expressing cells (IL-17^+^IFN-γ^−^: IL-17 SP, IL-17^−^IFN-γ^+^: IFN-γ SP) in CD4^+^YFP^+^ cells. **d**, **e** Naive CD4^+^ T cells were isolated from WT (CD45.1^+^CD45.2^+^) and *Tgfb1*^*fl/fl*^*Il17a*^*Cre*^*R26*^*YFP*^ (CD45.2^+^) mice, mixed at a 1:1 ratio and adoptively transferred into *Rag1*^*−/−*^ mice. **d** Schematic representation of T cell cotransfer colitis. **e** Flow cytometric analysis of IFN-γ and IL-17 expression in donor CD4^+^YFP^+^ cells (*n* = 7). Data are representative of at least two independent experiments. Quantification plots show the mean + (black) or – (red) SEM (**a**), + SEM (**b**; right graph), and ± SD (**c**); *p < 0.05, **p < 0.01, and ***p < 0.001. A two-tailed Student’s *t*-test (**a**–**c**) and the Wilcoxon signed-rank test (**e**) were performed.

**Fig. 6:**
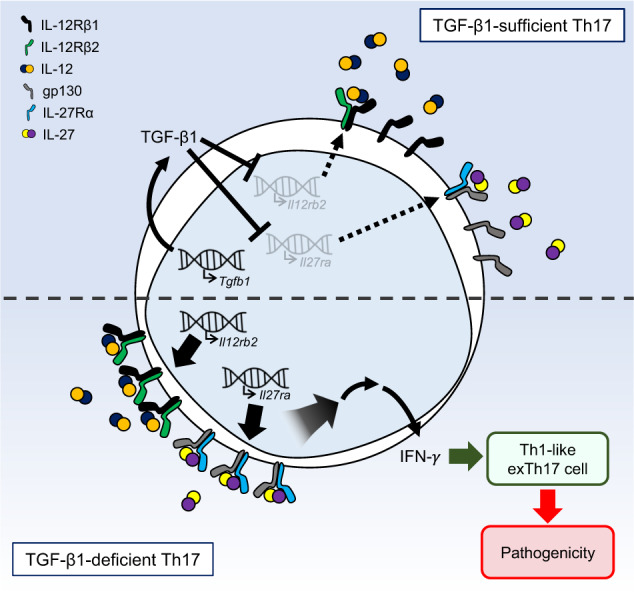
Schematic model of the proposed mechanism. TGF-β1 produced by Th17 cells represses the expression of *Il12rb2* and *Il27ra*, leading to reduced surface expression of receptors for IL-12 and IL-27. In the absence of Th17 cell-derived TGF-β1, Th17 cells express increased levels of *Il12rb2* and *Il27ra*, becoming more sensitive to IL-12 and IL-27 signals. Thus, TGF-β1-deficient Th17 cells are more prone to becoming IFN-γ producers, which are more pathogenic in inducing autoimmune inflammation in the CNS and gut. Hence, autocrine TGF-β1 from Th17 cells is critical for maintaining Th17 cell stability and for limiting the pathogenicity of these cells.

To further explore the role of Th17 cell-derived TGF-β1 in the regulation of Th17 stability and pathogenicity in different inflammatory settings, we employed an animal model of CD4^+^ T cell-mediated intestinal inflammation. CD4^+^CD25^−^CD44^low^CD62L^high^ naïve CD4^+^ T cells were isolated from both *Tgfb1*^*fl/fl*^*Il17a*^*Cre*^*R26*^*YFP*^ mice and WT mice and transferred into *Rag1*^*−/−*^ mice. While the recipients of naïve WT CD4^+^ T cells did not develop clinical signs of intestinal inflammation until 41 days after the cell transfer in our experimental model, the recipients of naïve *Tgfb1*^*fl/fl*^*Il17a*^*Cre*^*R26*^*YFP*^ CD4^+^ T cells started losing weight as early as day 20 after the transfer (Fig. 5a). Furthermore, the colon lengths of the mice in the latter group were significantly shorter than those of the mice in the former group (Fig. 5b). Consistent with the results from the EAE experiments shown in Fig. 4e and f, the frequency of IL-17^-^IFN-γ^+^ cells among YFP^+^ cells in the mesenteric LNs were significantly higher in the *Tgfb1*^*fl/fl*^*Il17a*^*Cre*^*R26*^*YFP*^ group (Fig. 5c). In contrast, the frequencies of Foxp3^+^ Treg cells in the mesenteric LNs were comparable between the two groups, indicating that Th17 cell-derived TGF-β1 did not impact the induction of Treg cells in this experimental model (Fig. S4). To directly compare the stability of TGF-β1-deficient and TGF-β1-sufficient Th17 cells within the same environment, we adoptively transferred a 1:1 mixture of naïve T cells isolated from *Tgfb1*^*fl/fl*^*Il17a*^*Cre*^*R26*^*YFP*^ mice (CD45.2^+^) and WT mice (CD45.1^+^CD45.2^+^) into *Rag1*^*−/−*^ mice (Fig. 5d). As shown in Fig. 5e, the frequency of IL-17^−^IFN-γ^+^ cells among YFP^+^ cells in the mesenteric LNs was significantly increased in the TGF-β1-deficient YFP^+^ donor cells compared to the TGF-β1-sufficient YFP^+^ donor cells. Consequently, we concluded that Th17 cell-derived TGF-β1 regulates the stability of Th17 cells and inhibits the pathogenic conversion of Th17 cells into Th1-like exTh17 cells by regulating the expression of IL-12Rβ2 and IL-27Rα during CNS or intestinal inflammation in vivo (Fig. 6).

## Discussion

During the differentiation of CD4^+^ T cells into effector cells, the expression of distinct and mutually exclusive transcription factors and establishment of epigenetic modifications allow committed Th cells to be stable. Nevertheless, fully differentiated effector Th cells are known to be plastic and convert into a different Th subset(s) in vivo. In particular, Th17 cells are known to become IFN-γ-producing Th17 cells or Th1-like exTh17 cells under lymphopenic or inflammatory conditions in vivo. How the stability and pathogenic conversion of Th17 cells are regulated remains incompletely understood. In this context, our present study unveiled a critical contribution of Th17 cell-derived TGF-β1 to the stability of Th17 cells by demonstrating that (i) autocrine TGF-β1 was required for the expression of latent TGF-β1 on Th17 cells, (ii) TGF-β1-deficient Th17 cells expressed increased levels of *Il12rb2* and *Il27ra* and were more prone to becoming Th1 cells, (iii) TGF-β1-deficient Th17 cells exacerbated autoimmune CNS inflammation and (iv) naïve *Tgfb1*^*fl/fl*^*Il17a*^*Cre*^*R26*^*YFP*^ CD4^+^ T cells induced more severe experimental colitis than naïve WT CD4^+^ T cells. Based on these findings, we propose that autocrine TGF-β1 critically contributes to the maintenance of Th17 cell stability by downregulating IL-12Rβ2 and IL-27Rα, allowing these cells to be more resistant to pathogenic conversion in autoimmune inflammation in vivo.

The role of autocrine TGF-β1 in the expression of LAP in Th17 cells is well supported by a previous study showing that fully differentiated Th17 cells express TGF-β1^23^. However, unlike *Tgfb1*^*fl/fl*^*Tnfrsf4*^*Cre*^ mice, *Tgfb1*^*fl/fl*^*Il17a*^*Cre*^ mice do not show any defects in the early differentiation of Th17 cells in vitro or in vivo^23,28^. Rather, TGF-β1 derived from Th17 cells may play an essential role in the maintenance of the Th17 cell lineage program. Mechanistically, autocrine TGF-β1 protects Th17 cells from Th1-converting cytokine signals by suppressing the expression of *Il27ra* and *Il12rb2*. In this context, our recent study demonstrated that autocrine TGF-β1 maintains the stability of Treg cells by inhibiting the expression of *Il12rb2* in Treg cells^29^. Thus, we propose a universal role for autocrine TGF-β1 in the maintenance of the lineage stability of both Treg cells and Th17 cells. While cotransfer experiments revealed a critical role for autocrine TGF-β1 in the maintenance of Th17 cell stability in intestinal inflammation and CNS autoimmunity, we could not rule out the possibility that Th17 cell-derived TGF-β1 also regulates the plasticity and pathogenicity of adjacent Th17 cells in a paracrine manner in vivo.

How does autocrine TGF-β1 regulate *Il12rb2* and *Il27ra* expression in Th17 cells? Previous studies have shown that STAT1 activation induces T-bet and subsequent IL-12Rβ2 expression in CD4^+^ T cells^30^. TGF-β1 has been shown to regulate the expression of T-bet by inhibiting IFN-γ-induced STAT1 phosphorylation^31^. Thus, it is possible that TGF-β1 regulates the expression of IL-12Rβ2 by inhibiting the STAT1-T-bet-IL-12Rβ2 pathway. Although TCR stimulation is known to induce IL-27Rα expression in CD4^+^ T cells^32^, less is known about the factors that induce or regulate IL-27Rα in CD4^+^ T cells. Considering that TGF-β1 inhibits the CD28-dependent growth and proliferation of CD4^+^ T cells via the Smad3 signaling pathway, it is possible that the TGF-β1-Smad3 pathway regulates IL-27Rα expression in CD4^+^ T cells by inhibiting T cell activation. The exact molecular mechanism by which autocrine TGF-β1 regulates IL-12Rβ2 and IL-27Rα in Th17 cells remains to be determined.

IL-27 induced more IFN-γ production than IL-12 when MOG-specific Th17 cells were restimulated, while IL-12 induced a higher frequency of IFN-γ producers in our experimental setting. Since IL-27 can induce IL-10 production in precommitted Th17 cells by inducing Blimp1^33^, it is possible that IL-10 produced late during restimulation of IL-17fm-YFP^+^ cells with IL-27 inhibited IFN-γ production by the IL-17fm-YFP^+^ cells. Further studies will be needed to address the molecular mechanism underlying the observed differential roles of IL-12 and IL-27 in IFN-γ production by Th17 cells.

Adoptive transfer experiments and fate-mapping studies revealed that autoreactive Th17 cells transdifferentiate into Th1-like Th17 cells or Th1-like exTh17 cells under inflammatory conditions. By utilizing LoxP/Cre-mediated deletion of specific genes during the late phase of Th17 differentiation in vivo, we and others have demonstrated that a large proportion of the Th17 cells found in the inflamed tissues of autoimmune mice are Th1-like exTh17 cells^10,12^. Consistently, Hirota et al.^10^ reported that the majority of IFN-γ-producing CD4^+^ T cells found in the inflamed CNS originated from Th17 cells. However, the physiological significance of Th1-like Th17 cells and Th1-like exTh17 cells in the pathogenesis of autoimmunity has been controversial^6,34–39^. Although T-bet was shown to be required for the generation of Th1-like Th17 cells or Th1-like exTh17 cells in several autoimmune disease settings, adoptive transfer of T-bet-deficient Th17 cells induced attenuated or comparable disease compared to transfer of WT Th17 cells depending on the experimental model^34–39^. Employing a more physiologically relevant animal model in which T-bet is selectively deleted in Th17 cells during the late phase of their differentiation in vivo, a recent study demonstrated that T-bet is required for the development of Th1-like exTh17 cells but not for Th17-mediated autoimmune immunopathology during intestinal inflammation and CNS inflammation^37^. In contrast, T-bet or IFN-γ production by Th17 cells^38,39^, in addition to IL-17 itself^40–42^, was found to be essential for the induction of intestinal inflammation^38^. Several more recent studies have demonstrated that the induction of Th1-like exTh17 cells is tightly associated with the development of autoimmune diseases^13–15^. Consistent with these findings, our study revealed the importance of Th17 cell plasticity in the pathogenesis of autoimmune diseases by showing that a deficiency in autocrine TGF-β1 in Th17 cells promotes Th17 cell-dependent intestinal or CNS inflammation. Given that Th1-like Th17 cells or Th1-like exTh17 cells have been found in the inflammatory regions of autoimmune patients^6,43,44^, the importance of Th17 cell plasticity in the pathogenesis of autoimmune diseases remains to be explored in a more physiologically relevant experimental setting.

A recent study identified two novel subsets of myelin-reactive Th17 cells, the stem-like CD27^+^TCF-1^hi^ subset, and the Th1-like CD27^-^T-bet^hi^ subset, in the dLNs of MOG_35-55_-immunized Th17 cell fate mapping mice^13^. Transcriptome analysis revealed that the CD27^+^TCF-1^hi^ subset of Th17 cells was enriched for stemness-associated signatures, while the CD27^-^T-bet^hi^ subset of Th17 cells was enriched for effector-associated signatures. In addition, after ex vivo MOG_35-55_ peptide restimulation, CD27^+^TCF-1^hi^ cells proliferated and then were converted into a CD27^−^T-bet^hi^ subset by the metabolic regulator mTORC1. Consequently, Th17 cell-specific deficiency in mTORC1 function protected mice from MOG-induced EAE. Since TGF-β1/Smad3 signaling has been reported to suppress mTORC1 in CD4^+^ T cells and NK cells^31,45^, it will be interesting to determine whether autocrine TGF-β1 regulates the transition between CD27^+^TCF-1^hi^ and CD27^-^T-bet^hi^ subsets by inhibiting mTORC1 in fully differentiated Th17 cells.

In summary, the present study reveals a previously unappreciated role for Th17 cell-derived TGF-β1 in the stability and pathogenic conversion of Th17 cells by using animal models of autoimmune tissue inflammation. Our findings provide molecular insights into the pathogenesis of T cell-mediated autoimmunity. Blockade of the pathogenic conversion of Th17 cells may pave the way for novel therapeutic strategies for the treatment of autoimmune diseases.

## Materials and methods

### Mice

C57BL/6 mice were purchased from Orient Bio (Seongnam, Gyeonggi-do, Republic of Korea). B6.SJL, *Tcrb*^*−/−*^, *Rag1*^*−/−*^, and *Il17a*^*Cre*^ mice were purchased from The Jackson Laboratory (Bar Harbor, ME, USA). *Tgfb1*^*fl/fl*^ mice were kindly provided by Dr. Ming O. Li (Memorial Sloan Kettering Cancer Center, New York, NY, USA). *R26*^*YFP*^ mice were kindly provided by Dr. Eric Vivier to Dr. Chang-Yuil Kang (Aix‐Marseille Université, Marseille, France)^46^. *Tgfb1*^*fl/fl*^*Il17a*^*Cre*^ mice were crossed with *R26*^*YFP*^ mice to generate *Tgfb1*^*fl/fl*^*Il17a*^*Cre*^*R26*^*YFP*^ mice. All mice were used at 8–10 weeks of age and maintained in the Animal Center for Pharmaceutical Research of Seoul National University under specific pathogen-free conditions.

### MOG immunization

C57BL/6, *Tgfb1*^*fl/fl*^*Il17a*^*Cre*^*R26*^*YF*P^, and WT (*Tgfb1*^*fl/+*^*Il17a*^*Cre*^*R26*^*YFP*^ or *Il17a*^*Cre*^*R26*^*YFP*^) control mice were subcutaneously immunized with 300 μg of myelin oligodendrocyte glycoprotein peptide (MOG_35-55_) (RS Synthesis, Louisville, KY, USA) emulsified in CFA (Sigma-Aldrich, St. Louis, MO, USA) with heat-killed *Mycobacterium tuberculosis* (BD Biosciences, San Jose, CA, USA). Eight to nine days after immunization, lymphoid cells from the dLNs were stained and analyzed by flow cytometry.

### In vitro Th17 cell differentiation

CD4^+^ T cells were isolated from the lymph nodes and spleen of *Tgfb1*^*fl/fl*^*Il17a*^*Cre*^*R26*^*YF*P^ and WT control mice with the CD4^+^ T Cell Isolation Kit (Miltenyi Biotec, Bergisch Gladbach, Germany), and then naïve CD4^+^ T cells (CD4^+^CD25^−^CD44^low^CD62L^high^) were sorted using a BD FACSAria^TM^ III (BD Biosciences, San Jose, CA, USA). Anti-CD3ε (145-2C11, 1 µg/mL) (BioXCell, West Lebanon, NH, USA) and anti-CD28 (37.51, 1 µg/mL) (BioXCell) Abs were used to precoat a 96-well flat-bottom plate (Corning, Steuben Country, NY, USA) overnight at 4 °C. After washing the plate with PBS, naïve CD4^+^ T cells (1 × 10^5^ cells/well) were cultured with recombinant mouse IL-6 (10 ng/mL) (PeproTech, Rocky Hill, NJ, USA) and recombinant human TGF-β1 (1 and 5 ng/mL) (PeproTech) for 72–96 h. The cultured cells were treated with 100 ng/mL PMA (Sigma-Aldrich, Saint Louis, MO, USA), 1 µM ionomycin (Sigma-Aldrich), brefeldin A (Thermo Fisher Scientific, Waltham, MA, USA), and monensin (Thermo Fisher Scientific) for an additional 3–6 h before flow cytometric analysis.

### Induction of myelin-reactive Th17 cells ex vivo and the adoptive transfer EAE model

Lymphoid cells were obtained from the dLNs of MOG_35-55_ peptide-immunized *Tgfb1*^*fl/fl*^*Il17a*^*Cre*^*R26*^*YF*P^ or WT control mice and restimulated with the MOG_35-55_ peptide (25 µg/mL) in the presence of recombinant mouse IL-23 (20 ng/mL) (Thermo Fisher Scientific) and an anti-IFN-γ antibody (XMG1.2, 5 µg/mL) (BioXCell) ex vivo to enrich MOG_35-55_-specific Th17 cells. After 5 days of stimulation, cytokine production in CD4^+^YFP^+^ T cells was analyzed by flow cytometry after PMA/ionomycin stimulation in the presence of brefeldin A and monensin. To induce EAE by adoptive transfer, CD4^+^ T cells were isolated from the MOG_35-55_-specific Th17 cell culture with the CD4^+^ T Cell Isolation Kit 5 days after stimulation. CD4^+^YFP^+^ T cells further purified on a FACSAria^TM^ III were adoptively transferred into *Tcrb*^*−/−*^ mice via tail vein injection (5 × 10^6^ cells/injection). One day after transfer, recipient mice were immunized with MOG_35-55_ peptide in CFA and then given an intraperitoneal injection of pertussis toxin (PTX, 500 ng/injection) (List Biological Laboratories, Campbell, CA, USA)^12^. Body weight and clinical disease score were monitored daily. Sixteen days after cell transfer, all mice were euthanized, and the dLNs, brain, and spinal cord were obtained for further analysis. In some experiments, CD4^+^YFP^+^ T cells from *Tgfb1*^*fl/fl*^*Il17a*^*Cre*^*R26*^*YF*P^ (CD45.2^+^) or WT control (CD45.1^+^CD45.2^+^) mice were mixed at a 1:1 ratio before transfer into *Tcrb*^*−/−*^ mice.

### Adoptive T cell transfer colitis model

Purified naïve CD4^+^ T cells (5 × 10^5^ cells) isolated from either *Tgfb1*^*fl/fl*^*Il17a*^*Cre*^*R26*^*YF*P^ mice or WT control mice were adoptively transferred into *Rag1*^*−/−*^ mice. In the mixed T cell transfer experiment, naïve CD4^+^ T cells (5 × 10^5^ cells) from WT control (CD45.1^+^CD45.2^+^) mice and *Tgfb1*^*fl/fl*^*Il17a*^*Cre*^*R26*^*YF*P^ (CD45.2^+^) mice were mixed at a 1:1 ratio before transfer into *Rag1*^*−/−*^ recipient mice. Recipients were monitored weekly for weight loss for 41 days. Mice were euthanized, and the colon was removed. Colon length was measured after sacrifice. The mesenteric lymph nodes were obtained for further flow cytometric analysis.

### Th17 cell conversion assay

For the Th17 cell conversion study, FACS-purified CD4^+^YFP^+^ cells (1 × 10^5^ cells/well) isolated from an in vitro Th17 cell differentiation culture were stimulated with recombinant mouse IL-12 (40 ng/mL) (PeproTech), recombinant mouse IL-27 (40 ng/mL) (R&D Systems, Minneapolis, MN, USA) and recombinant mouse IL-23 (20 ng/mL) in 96-well plates precoated with anti-CD3ε (1 µg/mL) and anti-CD28 (1 µg/mL) antibodies. After 3 days of culture, cells were treated with PMA, ionomycin, brefeldin A, and monensin for an additional 3–6 h before flow cytometric analysis.

### Flow cytometry

For CD4^+^ T cell analysis, cells from mice were stained with BUV737-conjugated anti-mouse CD4 (RM4-5, BD Biosciences), APC/Cy7-conjugated anti-mouse CD45.1 (A20, BioLegend, San Diego, CA, USA), PerCP/Cy5.5-conjugated anti-mouse CD45.1 (A20, BioLegend), BUV395-conjugated anti-mouse-CD45.2 (104, BD Biosciences), APC/Cy7-conjugated anti-mouse CD45.2 (104, BioLegend), PE/Cy7-conjugated anti-mouse-CD90.2 (Thy-1.2) (53-2.1, BioLegend), Pacific Blue^TM^-conjugated anti-mouse-CD90.2 (Thy-1.2) (53-2.1, BioLegend), PerCP/Cy5.5-conjugated anti-mouse-CD3ε (145-2C11, BioLegend), BUV395-conjugated anti-mouse-CD3ε (145-2C11, BD Biosciences), PE/Cy7-conjugated anti-mouse-CD44 (IM7, BioLegend), PE-conjugated anti-mouse LAP (TGF-β1) (TW7-16B4, BioLegend), and APC-conjugated anti-mouse LAP (TGF-β1) (TW7-16B4, BioLegend) antibodies. For intracellular staining, cells were fixed and permeabilized using the eBioscience^TM^ Intracellular Fixation & Permeabilization Buffer Set (Thermo Fisher Scientific) or Foxp3/Transcription Factor Staining Buffer Set (Thermo Fisher Scientific) and were stained with PE-conjugated anti-mouse LAP (TGF-β1) (TW7-16B4, BioLegend), APC-conjugated anti-mouse LAP (TGF-β1) (TW7-16B4, BioLegend), eFluor450-conjugated anti-mouse Foxp3 (FJK-16s, Thermo Fisher Scientific), Alexa Fluor^TM^ 647-conjugated anti-mouse Foxp3 (MF-14, BioLegend), PE-conjugated anti-mouse IFN-γ (XMG1.2, BioLegend), PE/Cy7-conjugated anti-mouse IFN-γ (XMG1.2, BioLegend), BUV395-conjugated anti-mouse IL-17A (TC11-18H10, BD Biosciences), and PE-conjugated anti-mouse IL-17A (TC11-18H10.1, BioLegend) antibodies. Cells were analyzed on an LSRFortessa^TM^ or a FACSLyric^TM^ (BD Biosciences), and the obtained data were analyzed using FlowJo software (BD Biosciences).

### Quantitative RT-PCR

To investigate gene expression in Th17 cells, we isolated total RNA from FACS-sorted MOG_35-55_-specific CD4^+^YFP^+^ cells using TRIzol^TM^ Reagent (Thermo Fisher Scientific) and synthesized cDNA from the isolated RNA with the RevertAid First Strand cDNA Synthesis Kit (Thermo Fisher Scientific) according to the manufacturer’s instructions. Relative gene expression levels were measured using iTaq Universal SYBR Green Supermix (Bio-Rad, Hercules, CA, USA) and an Applied Biosystems 7500 Fast real-time PCR system (Thermo Fisher Scientific). Primers for mouse *Actb* (forward: 5′-TGG AAT CCT GTG GCA TCC ATG AAA C-3′, reverse: 5′-TAA AAC GCA GCT CAG TAA CAG TCC G-3′), *Tgfb1* (forward: 5′-GCA ACA TGT GGA ACT CTA CCA GA-3′, reverse: 5′-GAC GTC AAA AGA CAG CCA CTC A-3′), *Tbx21* (forward: 5′-CAA CAA CCC CTT TGC CAA AG-3′, reverse: 5′-TCC CCC AAG CAG TTG ACA GT-3′), *Ifng* (forward: 5′-GAT GCA TTC ATG AGT ATT GCC AAG T-3′, reverse: 5′-GTG GAC CAC TCG GAT GAG CTC-3′), *Il17a* (forward: 5′-CTC CAG AAG GCC CTC AGA CTA C-3′, reverse: 5′-GGG TCT TCA TTG CGG TGG-3′), *Il12rb1* (forward: 5′-CCC CAG CGC TTT AGC TTT-3′, reverse: 5′-GCC AAT GTA TCC GAG ACT GC-3′), *Il12rb2* (forward: 5′-TGT GGG GTG GAG ATC TCA GT-3′, reverse: 5′-TCT CCT TCC TGG ACA CAT GA-3′), *Ifngr1* (forward: 5′-TCA AAA GAG TTC CTT ATG TGC CT-3′, reverse: 5′-TAC GAG GAC GGA GAG CTG TT-3′), *Ifngr2* (forward: 5′-TCC TGT CAC GAA ACA ACA GC-3′, reverse: 5′-ACA TCC AAT GTT GCT GCT GT-3′), *Il27ra* (forward: 5′-CAA GAA GAG GTC CCG TGC TG-3′, reverse: 5′-TTG AGC CCA GTC CAC CAC AT -3′) and *Il6st* (forward: 5′-ATA GTC GTG CCT GTG TGC TTA-3′, reverse: 5′-GGT GAC CAC TGG GCA ATA TG-3′) were purchased from Macrogen (Seoul, Republic of Korea) and Cosmogenetech (Seoul, Republic of Korea). Relative gene expression was normalized to the expression of β-actin (*Actb*).

### ELISA

IFN-γ in the culture supernatant of in vitro-differentiated Th17 cells stimulated under IFN-γ-inducing conditions was quantified using the IFN gamma Mouse Uncoated ELISA Kit (Thermo Fisher Scientific) as described in the manufacturer’s instructions.

### RNA-sequencing data information

Transcriptomic profiles of GSE135390, which consists of transcriptional analysis data for CD4^+^ T cell subsets from the blood of three donors, were downloaded from the GEO database (http://www.ncbi.nlm.nih.gov/geo/), and the *TGFB1* expression level was analyzed.

### Statistics

Data were analyzed with GraphPad Prism 8 (GraphPad Software, San Diego, CA, USA). *P* values were determined using a two-tailed Student’s *t*-test or the Wilcoxon signed-rank test and are presented within each figure and figure legend.

### Study approval

All animal experiments were performed according to protocols approved by the Institutional Animal Care and Use Committees of Seoul National University (protocol Nos. SNU-160422-3, SNU-191210-3, and SNU-181030-5).

## Supplementary information

Supplementary figures
