# Supplementary material for: A critical role for Th17 cell-derived TGF-β1 in regulating the stability and pathogenicity of autoimmune Th17 cells
Source: Exp Mol Med. 2021 May 28;53(5):993–1004. doi: 10.1038/s12276-021-00632-9 (PMC8178381; doi:10.1038/s12276-021-00632-9)
Supplement: Supplementary file 1 — Supplementary figures [file 12276_2021_632_MOESM1_ESM.docx]

**Supplementary information**

**
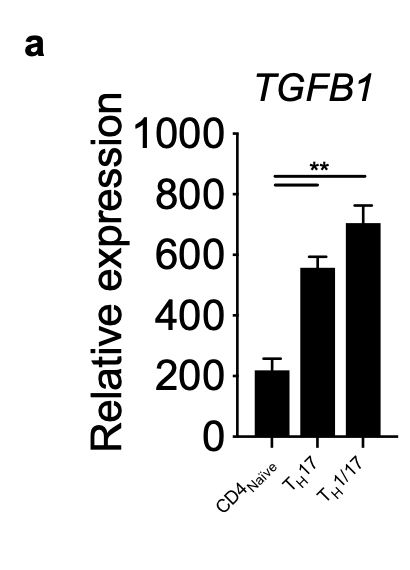
**

**Supplementary figure 1. *TGFB1* expression in human CD4^+^ T cell subsets.** Relative expression of *TGFB1* in human CD4^+^ T cell subsets (GSE135390). CD4_Naïve_ (CD127^+^CD25^-^CD45RA^+^), T_H_17 (CD127^+^CD25^-^CD45RA^-^CXCR3^-^CCR6^+^CCR4^+^CCR10^-^), and T_H_1/17 (CD127^+^CD25^-^CD45RA^-^CXCR3^+^CCR6^+^) were sorted from peripheral blood of healthy individual donors and the comprehensive transcriptional profiling were performed. Quantification plots show mean + SEM; ** *p* < 0.01. Two-tailed Student’s -test was performed.

**
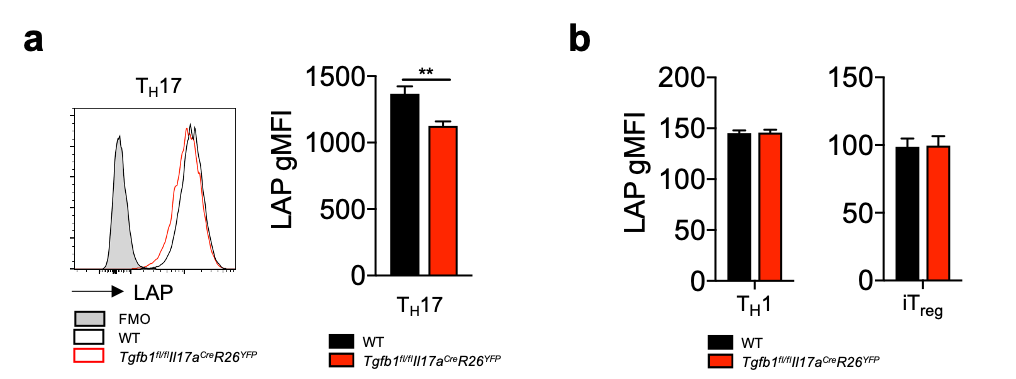
**

**Supplementary figure 2. Generation of IL-17-producing cell specific-TGF-β1-deficient mice. a** and **b** Naïve CD4^+^ T cells from *Tgfb1^fl/fl^Il17a^Cre^R26^YFP^* or wild-type (WT, *Il17a^Cre^R26^YFP^* or *Tgfb1^fl/+^Il17a^Cre^R26^YFP^*) mice were differentiated into Th1, Th17 or iTreg cells in vitro for three days and analyzed by flow cytometry. (a) Representative and geometric mean fluorescence intensity (gMFI) values of LAP among fluorescence minus one (FMO) control and gated IL-17^+^ cells (T_H_17) are shown. (b) Representative gMFI values of LAP among IFN-γ^+^ cells (T_H_1) or Foxp3^+^ cells (iT_reg_) are shown. Data are representative of three independent experiments values are expressed as the mean + SEM; ** *p* < 0.01. Two-tailed Student’s t-test was performed.

**
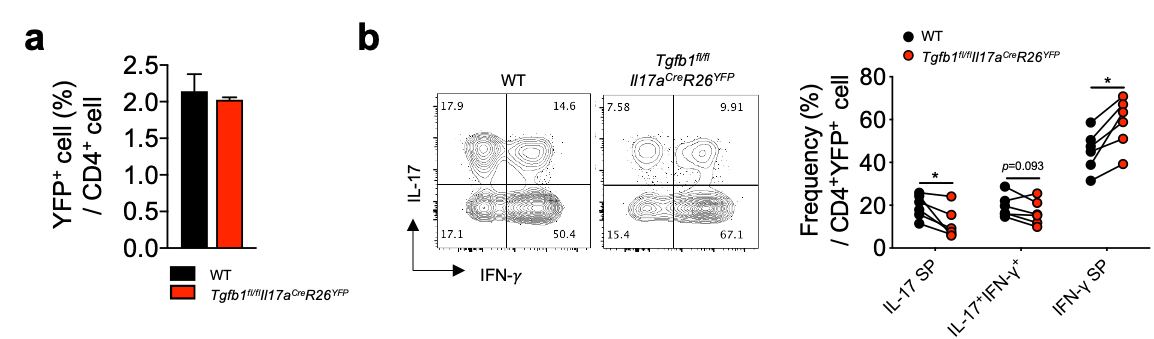
**

**Supplementary figure 3. Autocrine TGF-β1 maintains Th17 stability. a** *Tgfb1^fl/fl^Il17a^Cre^R26^YFP^* or WT mice were immunized with MOG_35-55_ peptide and analyzed after 9 days. Quantification of YFP^+^ cells among CD4^+^ cells in draining lymph nodes (dLNs). **b** TGF-β1-sufficient (CD45.1^+^CD45.2^+^) and TGF-β1-deficient (CD45.2^+^) myelin-reactive CD4^+^YFP^+^ Th17 cells were mixed at 1:1 ratio and adoptively transferred into *Tcrb^−/−^* mice followed by MOG_35-55_ / CFA immunization and PTX injection as described in Fig. 2e. Representative FACS plots and quantification of IFN-γ- and/or IL-17-expressing cells among CD4^+^YFP^+^ cells in central nervous system (*n* = 6). Data are representative of three independent experiments. Quantification plots show mean +SEM (a); * *p* < 0.05. Wilcoxon signed rank test was performed (b).

**
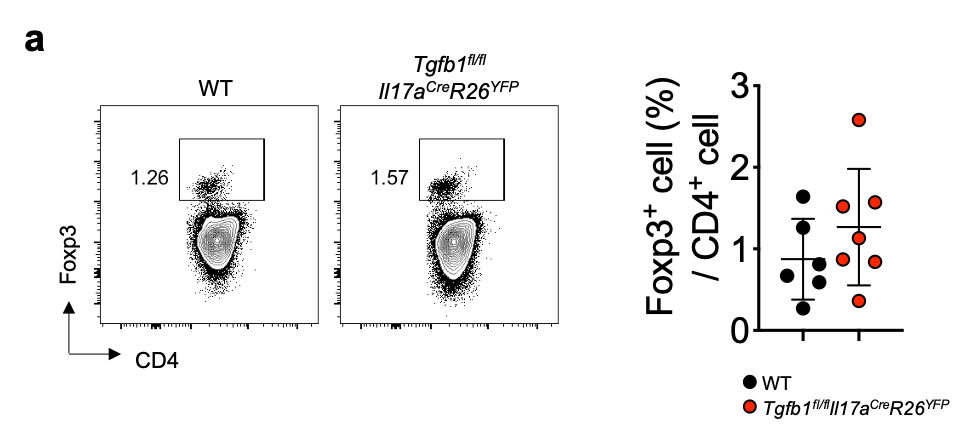
**

**Supplementary figure 4. Role of Th17-derived TGF-β1 in the development of induced Treg cells during intestinal inflammation.** Naïve CD4^+^ T cells were sorted and adoptively transferred into *Rag1^−/−^* mice as described in Fig. 5a. Flow cytometric analysis of Foxp3 expression among donor CD4^+^ cells in mesenteric LNs. Quantification plots show mean ± SD.
